# Special Issue: Ti-Based Biomaterials: Synthesis, Properties and Applications

**DOI:** 10.3390/ma13071696

**Published:** 2020-04-05

**Authors:** Jarosław Jakubowicz

**Affiliations:** Poznan University of Technology, Institute of Materials Engineering, Jana Pawla II no 24, 61-138 Poznań, Poland; jaroslaw.jakubowicz@put.poznan.pl; Tel.: +48-061-665-3571

**Keywords:** titanium, biomaterials, biofunctionalization

## Abstract

In the last half century, great attention has been paid to materials that can be used in the human body to prepare parts that replace failed bone structures. Of all materials, Ti-based materials are the most desirable, because they provide an optimum combination of mechanical, chemical and biological properties. The successful application of Ti biomaterials has been confirmed mainly in dentistry, orthopedics and traumatology. The Ti biomaterials provide high strength and a relatively low Young’s modulus. Titanium biocompatibility is practically the highest of all metallic biomaterials, however new solutions are being sought to continuous improve their biocompatibility and osseointegration. Thus, the chemical modification of Ti results in the formation of new alloys or composites, which provide new perspectives for Ti biomaterials applications. Great attention has also been paid to the formation of nanostructures in Ti-based biomaterials, which has leads to extremely good mechanical properties and very good biocompatibility. Additionally, the surface treatment applied to Ti-based biomaterials provides faster osseointegration and improve in many cases mechanical properties. The special issue “Ti-Based Biomaterials: Synthesis, Properties and Applications” has been proposed as a means to present recent developments in the field. The articles included in the special issue cover broad aspects of Ti-based biomaterials formation with respect to design theirs structure, mechanical and biological properties, as highlighted in this editorial.

Biomaterials, especially metal ones, are currently one of the most developed and used groups of materials. Titanium is the basic and one from the best biomaterial elements used in the pure form or as the main component in the biocompatible alloys or composites. Very good properties of titanium and its alloys are not able to cover up a few defects, which include for example chemical reactivity with atmospheric gases. Pure Ti, Ti alloys and Ti matrix composites are widely used in different fields. The titanium materials classification is based on structure at equilibrium state. Alloys of α, α + β and β structure are used in biomedical applications. The last one seems to be the most perspective, because theirs high biocompatibility and lowest among Ti alloys Young’s modulus, close to that of human bone, causes reduce stress shielding. Careful choice of chemical composition and processing parameters allow to design titanium biomaterials for most biomedical applications. The properties of Ti-based biomaterials should be as close as possible into the properties of the body element that the implant replaces. Hence the Young’s modulus is a key factor in biomaterials selection, preferring the titanium for hard tissue implants. Despite the very good properties of titanium and its alloys or composites, some properties are inadequate for biomedical device requirements. For example, wear resistance is poor and requires surface treatment. Deposition of more biocompatible surface layers provides safer used of implants. The nanocrystalline materials are a new group with interesting properties and a growing importance among of titanium biomaterials.

The special issue Ti-Based Biomaterials: Synthesis, Properties and Applications has been proposed to shows recent developments in the field of most extensively investigated and promising metallic biomaterials, and for this reason the nineteen articles included many different, however related aspects of Ti-based biomaterials. All the articles published in the special issue show the most important achievements in Ti-based biomaterials, useful for broader applications. A significant part of the articles included in the special issue focus on β-type Ti alloys, which are the most interesting among Ti alloys family. The β-type Ti alloys shows low modulus of elasticity and high biocompatibility, pointing the perspective and successful applications in hard tissue surgery. In the article by Bai et al. [1], the perspective β-type Ti-Mo alloys modified with Zr were presented. In the Ti-Mo-Zr system the diffusion coefficients were assessed to develop a self-consistent atomic mobility database of bcc phase. The calculated diffusion coefficients were compared with the experimental results showing good agreement. Sandu et al. [2] presents new β-type Ti-Mo alloys modified by Si, Zr and Ta, which shows very low Young’s modulus with perspective dental and orthopedic implant applications. Kuroda et al. [3] focus on the β-type Ti-Ta-Zr ternary alloys. The article shows combination of different processing routes (hot-rolling, annealing and solution treatment) for design of alloys of different mechanical properties (hardness and Young’s modulus). Fowler et al. [4] modified Ti-Nb-Ta-Zr alloy by addition of antibacterial Cu. As the effect of microstructural changes, the few % of Cu addition results in significant increase of alloys hardness. Consequently, in work [5], Fowler et al. continue study of Cu effect on antibacterial properties of Ti-Cu alloys. Generally, the Ti alloys had good antibacterial rates above 5 wt.% Cu content, however, the un-aged Ti-10 wt.% Cu alloy provide a good antibacterial effect, with superior hardness and corrosion protection. In addition to bulk β alloys, its porous form is very attractive for implant application. Formation of β-type Ti-Ta foams of 60%–76% porosity in the dealloying process was presented by Adamek et al. [6]. The nanocrystalline Ti-Ta-Mg powder mixture was prepared by mechanical alloying. Cold pressing followed by sintering at temperature above boiling point of Mg lead to its removal from the alloy matrix leaving the pores. The foams show mechanical and biological properties respective for hard tissue implant application.

Shape memory alloys are the second group of Ti-based materials with growing research interest in biomedical industry. Lee et al. [7] shows mechanical stability of the elastic nails made of the Nitinol shape memory alloy and compare them to titanium and stainless steel nails in the fixation of diaphyseal long bone fractures. The titanium nails have been recommended as C-shaped ones for diaphyseal long bone fractures when the end cap is not used. For Nitinol and stainless steel nails, use of the end cap prevent the nail from dropping out and provide bone fracture stabilization [7]. The Finite Element Method (FEM) model was compared with experimental results. The Nitinol was studied by Feng et al. [8], with respect to surface properties. The surface was modified using magnetic field-assisted Electrical Discharge Machining (EDM). Under the careful control of discharge conditions is generated a porous surface of different morphology and roughness, which finally effect on surface wettability. The main problem related with most commonly used Nitinols as shape memory alloys in medical industry is Ni as carcinogen. Nagoshi et al. [9] shows shape memory Ni-free β-type Ti-Nb alloys. The mechanical behavior of single crystalline micropillars was investigated in the microscale at various temperatures and crystal orientations to shows anisotropic deformation behavior and shape memory effect. The shape memory parts can work in biomedical microelectromechanical systems (Bio-MEMS).

Another group of Ti-based materials was presented by Miklaszewski and Jurczyk [10]. The Ti-TiB Metal Matrix Composites (MMC) were made using mechanical alloying of elemental Ti and B powders and pulse plasma sintering (PPS) for bulk sample generation. The Ti matrix is reinforced by TiB phase, which crystallizes during powders sintering in the PPS process. The processing conditions strongly effect on the structure and high mechanical properties of the ultrafine grains Ti-TiB composite.

New manufacturing methods are as often investigated as the Ti-based biomaterials themselves. In the work by Wei et al. [11], the Laser Additive Method (LAM) for the production of β-type Ti-Nb alloy has been shown. They used mixture of the elemental powders, which was directly melted under the laser beam. The additive technology in formation of alloy by melting of the powders mixture has many advantages over the melting prealloyed powders. The further annealing plays a key role for structure optimization and tailoring the mechanical properties. In the work by Bassoli and Denti [12], they focus on additive manufacturing (Laser Powder Bed Fusion—L-PBF) applied for synthesis Ti-6Al-4V for dental applications. The mechanical and structural properties were investigated, correlated and compared to those obtained for Co-Cr-Mo alloys. They found absence of secondary anisotropy in the L-PBF prepared specimens. Hsu et al. [13] applied Selective Electron Beam Additive Manufacturing (SEBAM) for the formation of Ti-6Al-4V under gradient energy density carried out on a specimen from the bottom to the top. This implies structural changes affecting the mechanical behavior.

According to Hall-Petch relation, the grain refinement leads to increase of materials strength. This relationship is used in Ti and its compounds. Different methods, based on severe plastic deformation (SPD) processes provide nano- or ultrafine structure of high strength and additionally good biocompatibility. Palán et al. [14] show properties of pure Ti after SPD processes (conform SPD and rotary swaging). They produced wires with ultrafine grains for medical implants. The process can be used in the large scale with low cost and the final products have ultimate strength around 1 GPa for Grade 2 cp-Ti.

Titanium shows good biocompatibility, however in some cases not sufficient for many applications. For the better osseointegration a different surface treatment is used for Ti parts surface modification. In the work by Elsayed et al. [15], the sphene (Titanite—CaTiSiO_5_) bioactive coatings were deposited on Ti substrate by airbrush spray technology using preceramic polymer with nano-sized precursors. The room temperature process deposition with subsequent heating results in crack-free coatings. The sphene coatings have good chemical stability, support hADSCs attachment, proliferation and differentiation in vitro. Authors point a potential for use of sphene bioactive coatings on orthopedic and dental implants. The surface treatment was undertaken by Benčina et al. [16] to generate TiO_2_ nanotubes on Ti substrate. The work describes crystallization process of the electrochemically made amorphous TiO_2_ nanotubes under a low pressure non-thermal oxygen plasma conditions. The surface can be potentially used for blood contacting devices because no platelet adhesion or activation on surfaces was observed. The biocompatibility improvement of the titanium maxillofacial plates treated with amine plasma-polymerization has been presented by Jeong et al. [17]. The Ti surface treatment undergoes through formation of polyallylamine ultra-thin film, which improved the hydrophilicity and biocompatibility confirmed in both in vitro and in vivo tests. The process applied for Ti surfaces can lead to shorten the time required for osseointegration and bone regeneration. In addition to the Ti surface modification, the Ti coatings can be deposited on different substrates for improvement their biocompatibility. In the work [18], Wypych et al. shows the Titanium Plasma Spraying (TPS) applied to polymer substrates. The Ti coatings exhibit a typical for TPS multilayer morphology of low porosity and good adhesion to polyethylene and glass fiber-reinforced polyamide. The Ti coatings did not fall off the substrate after its significant bending deformation. The TPS coatings have significantly greater hardness and Young’s modulus in comparison to the properties of the polymer substrates.

Finally, having materials and technology, it is possible to produce a biodevice. In this case, the numerical simulations including FEM can be helpful and are one of the steps for successful biodevice implementations. Gruenwald et al. [19] pointed that Ti electrical conductivity presents a challenge for the electromagnetic transmission of data and power. In article [19], the authors proposed a fast and practical method to determine the necessary transmission parameters for titanium encapsulated implants with inductive transmission, for example implanted infusion pump (with an external controlling device). 

Summarizing, the special issue Ti-Based Biomaterials: Synthesis, Properties and Applications shows current achievements in Ti-based materials, pointing future research trends in this field of interest.
